# Healthy but not osteoarthritic human meniscus-derived matrix scaffolds promote meniscus repair

**DOI:** 10.3389/fbioe.2024.1495015

**Published:** 2024-10-29

**Authors:** Saman Firoozi, Jon C. Ley, Dawn A. D. Chasse, David E. Attarian, Samuel S. Wellman, Annunziato Amendola, Amy L. McNulty

**Affiliations:** ^1^ Department of Orthopaedic Surgery, Duke University School of Medicine, Durham, NC, United States; ^2^ Department of Pathology, Duke University School of Medicine, Durham, NC, United States; ^3^ Department of Biomedical Engineering, Duke University, Durham, NC, United States

**Keywords:** tissue engineering, proteoglycans, osteoarthritis, allograft, meniscus, age, collagen, meniscus repair

## Abstract

Meniscus tissue is commonly injured due to sports-related injuries and age-related degeneration and approximately 50% of individuals with a meniscus tear will develop post-traumatic osteoarthritis (PTOA). Given that the meniscus has limited healing potential, new therapeutic strategies are required to enhance meniscus repair. Porcine meniscus-derived matrix (MDM) scaffolds improve meniscus integrative repair, but sources of human meniscus tissue have not been investigated. Therefore, the objectives of this study were to generate healthy and osteoarthritic (OA) MDM scaffolds and to compare meniscus cellular responses and integrative repair. Meniscus cells showed high viability on both healthy and OA scaffolds. While DNA content was higher in cell-seeded OA scaffolds than cell-seeded healthy scaffolds, CCK-8, and both sGAG and collagen content were similar between scaffold types. After 28 days in an *ex vivo* meniscus defect model, healthy and OA scaffolds had similar DNA, sGAG, and collagen content. However, the shear strength of repair was reduced in defects containing OA scaffolds compared to healthy scaffolds. In conclusion, healthy human allograft tissue is a useful source for generating MDM scaffolds that can support cellular growth, ECM production, and *ex vivo* integrative repair of the meniscus, highlighting the potential suitability for tissue engineering approaches to improve meniscus repair.

## 1 Introduction

Menisci are fibrocartilaginous tissues located between the femoral condyles and tibial plateau in the knee. The menisci transfer and distribute load across the articular cartilage, provide joint stability, and afford a low friction surface for joint articulation (Fox et al., 2012; Ahmed et al., 1983; Markolf et al., 1981; Wojtys and Chan, 2005; Carter et al., 2015). Proper meniscus function is necessary for maintaining performance and health of the knee joint (Ahmed et al., 1983; Markolf et al., 1981; Wojtys and Chan, 2005; Carter et al., 2015). Due to joint loading during activities of daily living and sports, the menisci must resist high forces in tension and compression and thus are highly susceptible to injury (Vignes et al., 2022). The yearly incidence of meniscus injury is 66 tears per 100,000 people (Bansal et al., 2021). These tears can be primarily traumatic or degenerative due to sports-related injuries or age-related joint degeneration respectively (Terzidis et al., 2006; Englund et al., 2009). There is pain and disability associated with the meniscus injury but also nearly 50% of individuals with a meniscus tear develop post-traumatic osteoarthritis (PTOA) within 20 years of the injury (Blake and Johnson, 2018; Scotti et al., 2013; Lohmander et al., 2007; Badlani et al., 2013). Therefore, current orthopaedic treatments seek to restore meniscus structure and function through different approaches including meniscus repair, allograft transplantation, and biological augmentation strategies (Lin et al., 2017; Guo et al., 2015; Chevrier et al., 2018; Wang et al., 2024; Luvsannyam et al., 2022). Despite promising short-term results, these treatments fail to restore function of the injured meniscus tissue and are not able to prevent PTOA development (Scotti et al., 2013; Wang et al., 2024). Given that the meniscus has a limited healing potential due to its dense extracellular matrix (ECM) and minimal vascularity (Xia et al., 2021), new therapeutic strategies are required to enhance meniscus repair.

Meniscus tissue engineering is a promising approach to replace lost and/or damaged meniscus tissue. Various types of biomaterials, including natural and synthetic polymers, hydrogels, and tissue-derived materials, have been used to generate tissue-engineered scaffolds for meniscus regeneration (Makris et al., 2011; Li et al., 2021b; Ionescu and Mauck, 2013; Chen et al., 2019; Guo et al., 2021; Gao et al., 2017; Komatsu et al., 2024; An et al., 2021). Decellularized ECM is a promising choice because it contains the native components of the ECM, natural growth factors found in the matrix, and provides tissue specific epitopes to regulate cellular behavior and function (Hussey et al., 2018; Pati et al., 2014). To date, numerous forms of meniscus-derived matrix (MDM) scaffolds have been investigated including whole lyophilized tissue grafts (Gelse et al., 2017; Shimomura et al., 2017; Jiang et al., 2018), pulverized tissue reconstituted as porous or hydrogel scaffolds (Wu et al., 2015; Yuan et al., 2017; Ruprecht et al., 2019; Lyons et al., 2019; Xia et al., 2021; Salinas-Fernandez et al., 2024), 3D printed scaffolds composed of ECM-based bioinks (Pati et al., 2014; Ahn et al., 2017; Yang et al., 2017; Grogan et al., 2013; Bakarich et al., 2014; Lian et al., 2024; Wang et al., 2023), electrospun scaffolds (Baek et al., 2016; Venugopal et al., 2018; Mandal et al., 2011a; Li et al., 2021a), or a combination of these strategies (Baek et al., 2015; Yu et al., 2019). In our prior work, we showed that pulverized and reconstituted porcine MDM scaffolds can promote migration of endogenous meniscus cells and can improve the integrative repair of a porcine *ex vivo* meniscus defect (Ruprecht et al., 2019). For clinical applications, MDM can be isolated from allogeneic or xenogeneic meniscus tissues. However, there are concerns regarding the immunogenicity of xenogenic scaffolds for use in humans (Stone et al., 2017; Chen et al., 2024). Alternatively, there is limited availability of healthy human meniscus tissue. Prior work has shown that discarded human kidneys procured for transplantation were successfully used to generate ECM scaffolds for renal tissue engineering applications (Orlando et al., 2013). According to the 2024 report of the American College of Rheumatology, there are approximately 790,000 total knee joint replacement surgeries performed annually in the US (Ray, 2024), so there is a large abundance of human osteoarthritic (OA) menisci available as waste following surgery (Kremers et al., 2015). These tissues could be an abundant and useful source for human MDM scaffold fabrication.

Therefore, the objectives of this study were to generate healthy and OA MDM scaffolds and to compare *in vitro* meniscus cellular responses and meniscus integrative repair capacity of the scaffolds using an *ex vivo* human meniscus defect model. A new combination of physical and chemical methods was employed to fabricate decellularized healthy and OA scaffolds. Then, the response of human meniscus cells to healthy and OA scaffolds was evaluated using *in vitro* assays. Using a cell-free approach, healthy and OA scaffolds were implanted in an *ex vivo* human meniscus defect model to compare cellular migration of native cells, tissue formation, and integrative repair capacity of the healthy *versus* OA scaffolds.

## 2 Materials and methods

### 2.1 Human and OA meniscus samples

Deidentified OA human medial and lateral menisci (N = 31, 22F/9M) with a mean age of 65 ± 8.2 years old were obtained from subjects undergoing total knee replacement at Duke Hospital (IRB Pro00079807). Healthy human medial menisci (N = 10, 5F/5M) from donors with an average age of 25 ± 6 years old were generously donated by JRF Ortho. Photographs were taken of all menisci prior to storage at −80°C.

### 2.2 Gross grading

Gross grading (n = 5 healthy, n = 10 OA) was performed independently by four blinded graders from photographs and the results were averaged. The scoring criteria was previously published by Pauli et al. (2011). Briefly, each meniscus was divided into 3 zones and each zone was given a score between 1 and 4. The total for each zone was then summed, yielding a total of 3 for a normal intact meniscus and a maximum score of 12 for a meniscus with full/complete substance tears, loss of tissue, and/or tissue maceration in all zones.

### 2.3 Histological analyses

Healthy and OA menisci (n = 3/group) were sliced radially for cross-sectional analysis in the central portion of the menisci. These tissues and day 28 meniscus defect model explants (see Meniscus explant harvest and *ex vivo* meniscus defect below, n = 3/group) were fixed in formalin overnight at 4°C, dehydrated, and paraffin embedded. Then samples were cut into 8 μm sections and stained with Harris’ hematoxylin (Electron Microscopy Sciences, #26754-01, United States), 0.02% aqueous fast green (Electron Microscopy Sciences, #15500, United States), and 0.1% Safranin-O (Sigma-Aldrich, #HT904-8FOZ) (Ruprecht et al., 2019).

### 2.4 MDM scaffold fabrication and decellularization

**FIGURE 1:**
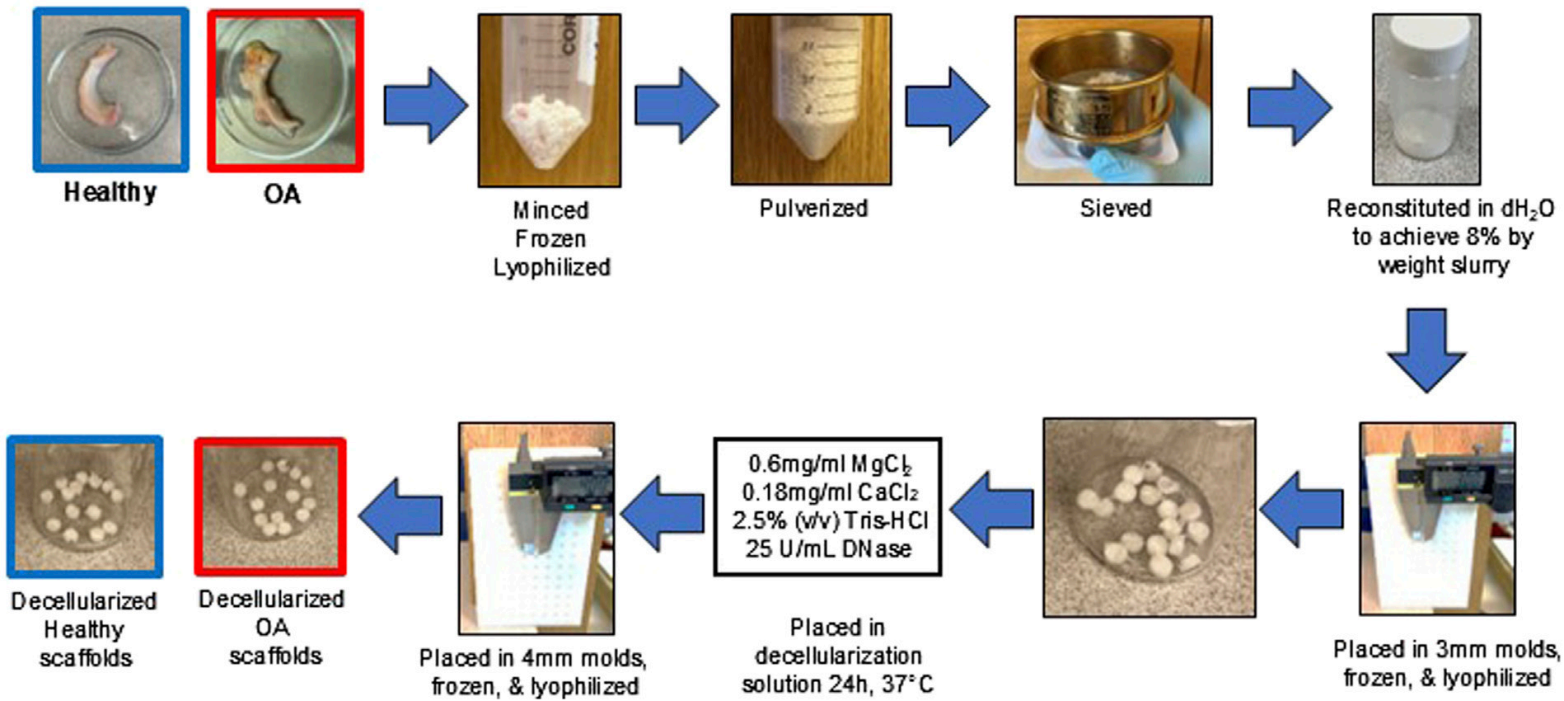
Fabrication procedure of healthy and OA MDM scaffolds.

The healthy and OA menisci were thawed, minced into ≤5 mm pieces, frozen overnight at −80°C, and then lyophilized (FreeZone 2.5 L, Labconco, Kansas City, MO) for 24 h (Figure 1). The lyophilized meniscus pieces were pulverized (5 min pre-cool, 5 cycles of 1 min at 5 Hz and 2 min cool) in a 6770 freezer mill (SPEX SamplePrep, Metuchen, NJ) (Ruprecht et al., 2019). The healthy and OA meniscus powders were sieved at 500 µm and pooled into two separate superlots. Healthy and OA powders were rehydrated to 8% weight fraction by mixing 0.8 g MDM powder with distilled water to achieve 10 g total mass. After resuspension, the healthy and OA slurry was homogenized (VWR^®^ 200 Homogenizer) at 30,000 rpm for 2 cycles of 2 min run then 2 min cool on ice (Rowland et al., 2016; Ruprecht et al., 2019; Lyons et al., 2019). Healthy and OA homogenized slurries were pipetted into delrin molds (1.9 mm deep × 3 mm diameter). Molds were then frozen at −80°C overnight and lyophilized for 24 h. Next, fabricated healthy and OA scaffolds were removed from the mold and incubated in decellularization solution composed of 0.6 mg/mL MgCl_2_ (Sigma-Aldrich, M8266), 0.18 mg/mL CaCl_2_ (Sigma-Aldrich, C3889), 2.5% (v/v) Tris-HCl (Sigma-Aldrich, T9285), and 25 U/mL DNase (Sigma-Aldrich, D5319) at 37°C for 24 h (Rowland et al., 2016). Scaffolds were gently placed into 4 mm diameter delrin molds using tweezers, frozen at −80°C overnight, lyophilized for 24 h, and kept in sterile containers. Both non-decellularized and decellularized scaffolds were washed 5 times with PBS prior to biochemical assessments and *in vitro* and *ex vivo* experiments.

### 2.5 Biochemical assays

In order to determine biochemical content of scaffolds before and after decellularization (n = 3–4/group), throughout *in vitro* culture (n = 3/group/timepoint), and following *ex vivo* culture (n = 18/group), meniscus inner cores and scaffolds were digested in 500 μL of papain (125 μg/mL papain (Sigma-Aldrich, P4762), 100 mM phosphate buffer (Sigma-Aldrich, S9638), 10 mM cysteine (Sigma-Aldrich, C1276), and 10 mM EDTA, pH 6.3 (Sigma-Aldrich, E7989) at 65°C overnight (Rowland et al., 2013; Collins et al., 2019; Hatcher et al., 2017). DNA content was measured by following the manufacturer’s instructions for the picogreen assay (Invitrogen, P7589) (Lyons et al., 2019; Ruprecht et al., 2019). The sGAG content was determined by the dimethylmethylene blue assay (Farndale et al., 1986), using a bovine chondroitin sulfate standard (Sigma-Aldrich, C6737). Collagen content was measured by the hydroxyproline assay (Reddy and Enwemeka, 1996; Collins et al., 2019; Hatcher et al., 2017; Lyons et al., 2019; Ruprecht et al., 2019).

### 2.6 Human meniscus cell isolation and scaffold cell seeding

Human meniscal cells were isolated from joint replacement waste meniscus tissue (N = 3, 2F/1M, 54 ± 22.5 years old). Immediately after surgical removal, menisci were placed in sealed containers with sterile saline. The whole meniscus was minced into ∼2 mm × 2 mm pieces and washed in Dulbecco’s modified eagle medium-high glucose (DMEM-HG, Gibco) containing 10% antibiotic–antimycotic (penicillin–streptomycin–fungizone (PSF), Gibco). Meniscus pieces were then enzymatically digested (Riera et al., 2011; McNulty et al., 2013) with 0.5% pronase (Calbiochem, San Diego, CA, United States) for 1 h followed by overnight digestion with 0.2% collagenase type I (Worthington, Lakewood, NJ, United States) in DMEM-HG containing 10% PSF and 10% fetal bovine serum (FBS; Corning, Corning, NY, United States) at 37°C (Andress et al., 2021; Andress et al., 2022). Isolated cells were then filtered through a 70 μm cell strainer (Corning). Isolated meniscus cells from three different patients were pooled together and expanded in meniscus growth medium consisting of DMEM-HG supplemented with 10% FBS, 1% PSF, 1% 4-(2-hydroxyethyl)-1-piperazine ethanesulfonic acid buffer (HEPES, Invitrogen, Carlsbad, CA, United States), 1% non-essential amino acids (Invitrogen), and 100 μg/mL ascorbic acid 2-phosphate (Sigma–Aldrich) at 37°C, 5% CO_2_. Passage 1 meniscus cells were trypsinized, counted, and resuspended in meniscus growth medium at a density of 7.8 × 10^6^ cells/mL (Lyons et al., 2019). Healthy and OA scaffolds were placed in ultra-low attachment 24 well plates (Corning). Then 8 μL of cell suspension was pipetted on the top of each scaffold. Scaffolds were placed into a vacuum chamber for 45 s to improve cell infiltration (Solchaga et al., 2006). Scaffolds were flipped and 8 μL of cells were pipetted onto the other side of the scaffolds and vacuum infiltration was repeated (Lyons et al., 2019). Then 1 mL of meniscus growth medium was added to each well and scaffolds were incubated at 37°C and 5% CO_2_ for up to 14 days. Growth media was changed every 2–3 days.

### 2.7 Evaluation of meniscus cellular responses on scaffolds

#### 2.7.1 Actin immunostaining

At 1, 3, 6, and 24 h after cell seeding, scaffolds were fixed in 4% paraformaldehyde (PFA, Electron Microscopy Sciences, 15,710) for 20 min to evaluate cellular attachment (n = 3/group/timepoint) as described previously (Xia et al., 2021). Scaffolds were subjected to antigen retrieval using the Universal antigen retrieval reagent (Abcam, ab208572) for 20 min. Next, samples were permeabilized with 0.5% Triton X-100 (Sigma-Aldrich) at RT for 30 min, blocked in 3% bovine serum albumin (Sigma-Aldrich, A9418) for 1 h at 37°C, and incubated overnight with 1 μg/mL recombinant anti-actin antibody (Abcam, ab213251) at 4°C. Then 1 μg/mL goat anti-human IgG-488 (Abcam, ab69907) was applied at 37°C for 1 h. Scaffolds were counterstained with DAPI (Invitrogen) and imaged using a fluorescent microscope (Olympus, IX83).

#### 2.7.2 Live/dead staining

On days 3 and 7, healthy and OA cell-seeded scaffolds were stained with the Live/Dead Assay kit (Invitrogen, L34973) to assess cell viability (n = 3/group/timepoint) (Van Der Straeten et al., 2016; Wilusz et al., 2008). Scaffolds were incubated with 2 mM calcein AM and 4 mM ethidium homodimer-1 for 30 min and then washed in PBS. Images were acquired using a fluorescent microscope (Olympus, IX83). Images were deconvoluted and live and dead cells were counted using cellSens Dimension version 1.18 software (Olympus, Center Valley, PA). Percent cell viability was calculated as follows: % Cell viability = (live cells/total cells) × 100.

#### 2.7.3 Edu staining

On days 3 and 13, fresh culture media was added containing 10 μM 5-ethylnyl-2′-deoxyuridine (EdU from the Click-iT™ EdU Alexa Fluor^®^ 488 Imaging Kit; Invitrogen C10337). After 24 h, on days 4 and 14, EdU staining was performed to detect proliferative cells on cell-seeded scaffolds, according to the manufacturer’s protocol (n = 3–4/group/timepoint). Samples were imaged using a fluorescent microscope (Olympus, IX83). Images were deconvoluted and the number of EdU-positive and total cells were counted using the cellSens Dimension version 1.18 software. The percent proliferative cells were calculated as follows: % Proliferative cells = (Edu^+^ cells/total cells) × 100.

#### 2.7.4 Biochemical assessment of cell viability

On days 1, 4, 7, and 14, cell counting kit-8 (CCK-8 kit, APExBIO, K1018) was used to evaluate cell viability within the healthy and OA scaffolds (n = 3/group/timepoint). Growth media containing CCK-8 solution at a final concentration of 10% was added to each cell-seeded scaffold (Chen et al., 2019). After 3 h at 37°C, the media was removed and read at 450 nm on a microplate reader (TECAN, Austria).

### 2.8 Meniscus explant harvest and *ex vivo* meniscus defect model

**FIGURE 2:**
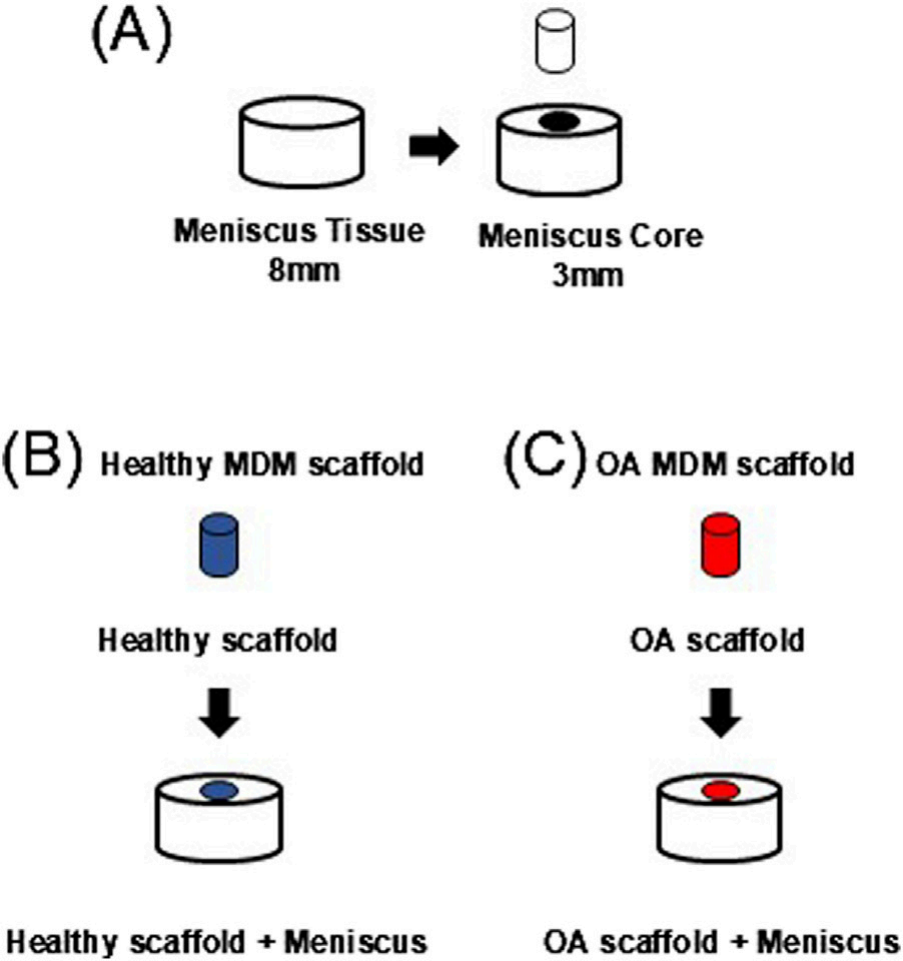
**(A)** Schematic of meniscal defect model. Implantation of **(B)** healthy and **(C)** OA scaffolds.

Waste human menisci (N = 11, 9F/2M, 69 ± 7.82 years old) from joint replacement surgeries were used to harvest meniscus defect model explants. For each subject, the grossly healthier meniscus (Pauli et al., 2011) was used. Tissue explants were harvested using 8 mm diameter biopsy punches and cut to 2 mm thick explants (Figure 2A). Then a 3 mm diameter core was removed from each explant to create a full-thickness defect (Hennerbichler et al., 2007a; McNulty et al., 2010; McNulty and Guilak, 2008; McNulty et al., 2007; McNulty et al., 2009; Ruprecht et al., 2019; Lyons et al., 2019). The defect was filled with either a healthy (Figure 2B, Healthy scaffold + Meniscus) or OA scaffold (Figure 2C, OA scaffold + Meniscus). All samples were cultured in meniscus growth media and incubated at 37°C and 5% CO_2_ for up to 28 days. Growth media was changed every 2–3 days.

### 2.9 Evaluation of meniscus defect model explants

#### 2.9.1 Fluorescent imaging of meniscus defect model explants

Meniscal defect model explants were harvested at days 7, 14, 21, and 28 (n = 3/group/timepoint) to visualize migration of cells from the outer meniscus tissue to the inner core by staining of live cells using calcein AM (Invitrogen, L3224) and matrix with Alexa fluor 633 NHS ester (Invitrogen, A20005) (Lyons et al., 2019; Ruprecht et al., 2019). A fluorescent microscope (IX83, Olympus) was used to visualize the cells and matrix.

#### 2.9.2 Shear strength of repair

After 28 days of culture, meniscus defect model explants were subjected to a push-out test to measure shear strength of repair (n = 12/group) (Hennerbichler et al., 2007a; McNulty et al., 2010; Ruprecht et al., 2019; Lyons et al., 2019; McNulty et al., 2007). Explants were loaded into a dish containing a central 4 mm hole and placed in the test frame (ElectroForce 3220 Series III, MN, United States). A 2 mm piston connected to a 225N load cell (Honeywell, Morris Plains, NJ) and centered directly above the inner core was used to push-out the inner core. A 0.5 g tare load was held for 10 s, and then the piston was displaced at a rate of 0.083 mm/sec to 5 mm. Images of each explant were collected and imported into ImageJ (NIH, United States) to measure sample height. Shear strength of repair was calculated as the peak force divided by the area of the repair interface. The displaced inner core scaffolds were papain digested for biochemical analyses (see Biochemical assays above).

### 2.10 Statistical analyses

Statistical analyses were performed using GraphPad Prism 10. All data was tested for normality. For gross grading and the *ex vivo* biochemical data, unpaired t-tests were performed. For decellularization DNA content and *in vitro* biochemical outcomes, two-way ANOVAs followed Tukey’s *post hoc* testing were performed. For decellularization matrix content, cell viability, and proliferation, two-way ANOVAs with Sidak’s multiple comparison testing was used. The data for the shear strength of repair was not normally distributed so Mann-Whitney U testing was performed. For all experiments *p* < 0.05 was considered statistically significant.

## 3 Results

### 3.1 Assessment of meniscus tissue

**FIGURE 3:**
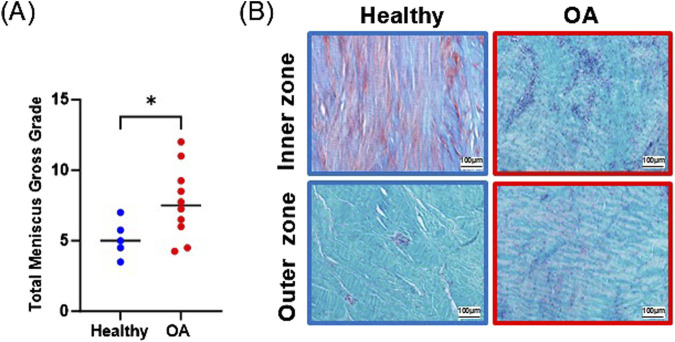
**(A)** Gross grading scores. The mean is indicated by a line for each group. *p < 0.05. **(B)** Safranin O and fast green staining of representative healthy and OA meniscus tissue.

Gross grading revealed that OA meniscus tissue had significantly higher scores than healthy meniscus tissue, indicative of degradative changes in the OA tissues (Figure 3A, *p* < 0.05). Histological staining of the healthy meniscus tissue showed sparce cells distributed throughout the tissue and there was strong proteoglycan staining in the inner zone (Figure 3B). On the other hand, in OA meniscus tissue, there was hypercellularity and diminished proteoglycan staining.

### 3.2 Evaluation of decellularization of scaffolds

**FIGURE 4:**
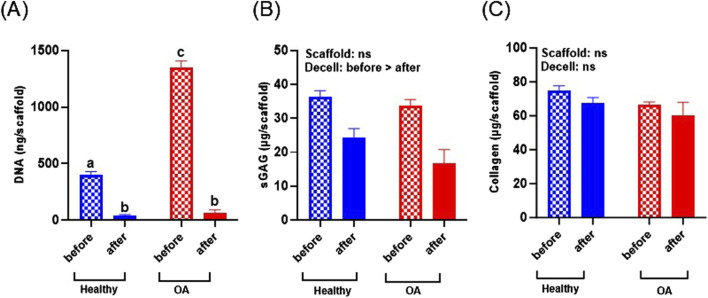
**(A)** DNA, **(B)** sGAG, and **(C)** collagen content of healthy and OA scaffolds before and after decellularization (Decell). Data are expressed as the mean +SEM for all groups. All groups not sharing the same letter have *p*-values <0.0001.

DNA content of the OA scaffolds was significantly higher than healthy scaffolds before decellularization (Figure 4A, *p* < 0.0001), indicating that the OA menisci contained more cells. Decellularization significantly decreased DNA content of both healthy and OA scaffolds (*p* < 0.0001). After decellularization, there were no detectable differences in the DNA content between the healthy and OA scaffolds. sGAG was not detectably different between scaffold types but was slightly reduced due to decellularization (Figure 4B, *p* < 0.05). On the other hand, collagen content was retained in both the healthy and OA scaffolds after decellularization and there were no detectable differences between the scaffold types (Figure 4C).

### 3.3 Meniscus cellular responses on scaffolds

**FIGURE 5:**
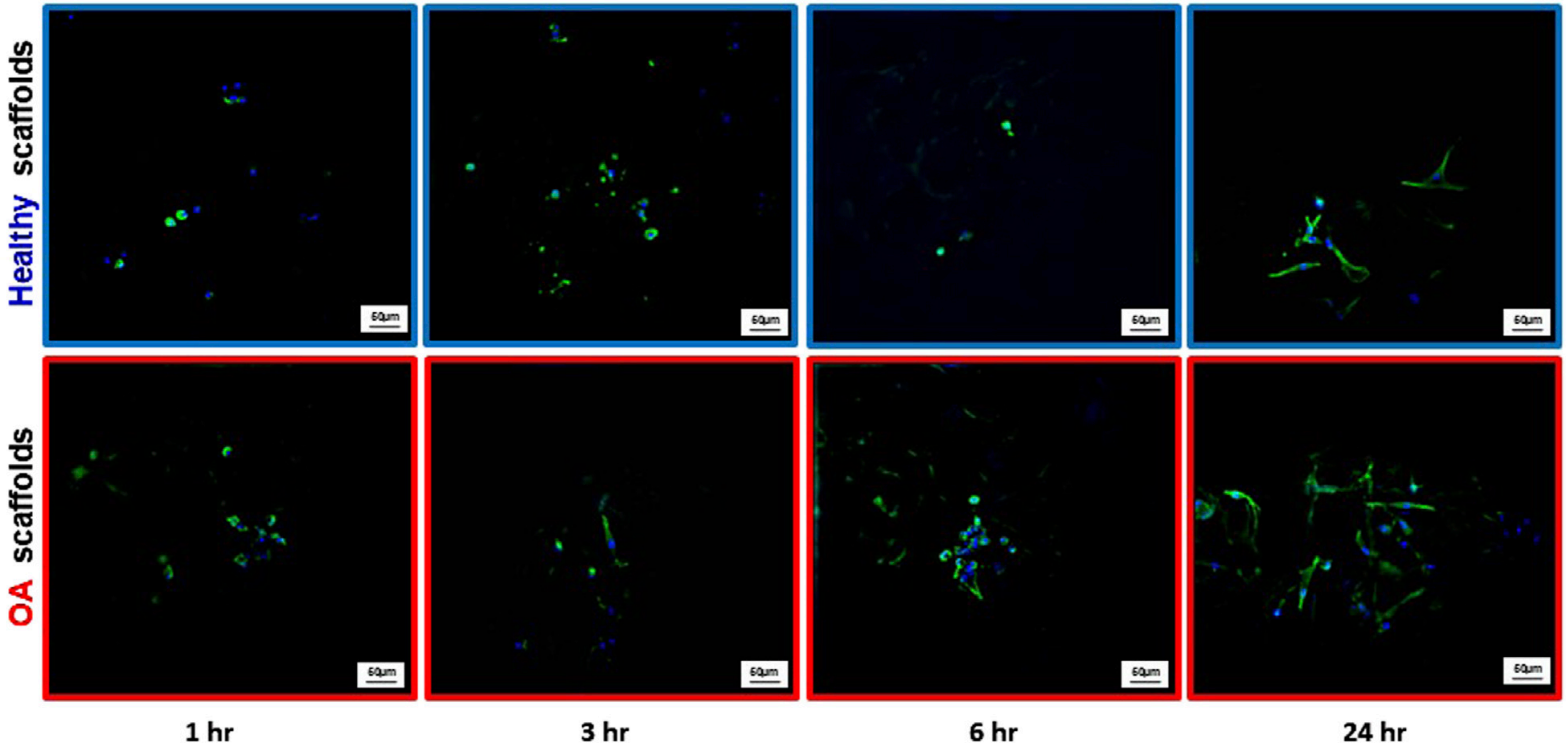
Representative images of actin (green) and DAPI (blue) staining of human meniscal cells seeded onto healthy and OA scaffolds.

**FIGURE 6:**
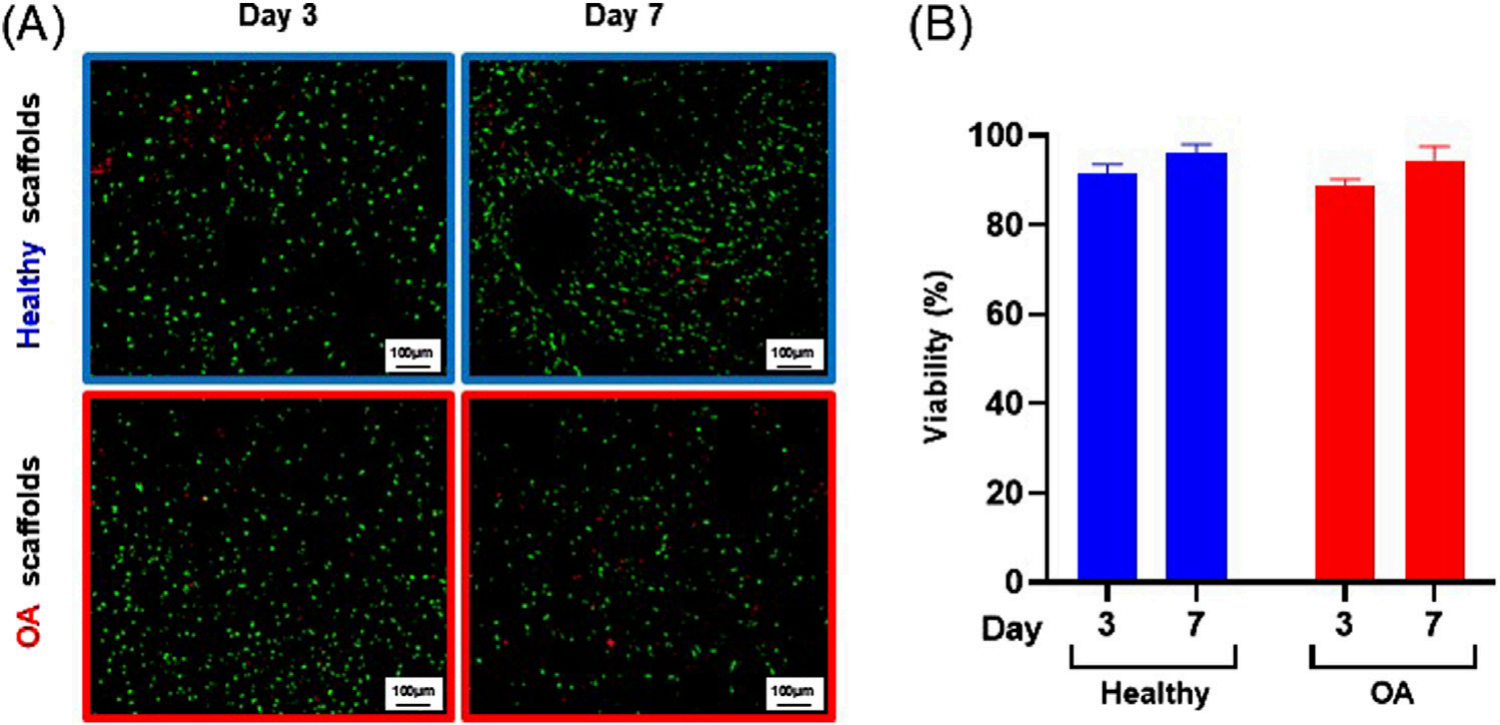
**(A)** Representative images of live (green) and dead (red) human meniscal cells seeded onto healthy and OA scaffolds. **(B)** Percent viability of cells on healthy and OA scaffolds. Data are expressed as the mean +SEM for all groups.

**FIGURE 7:**
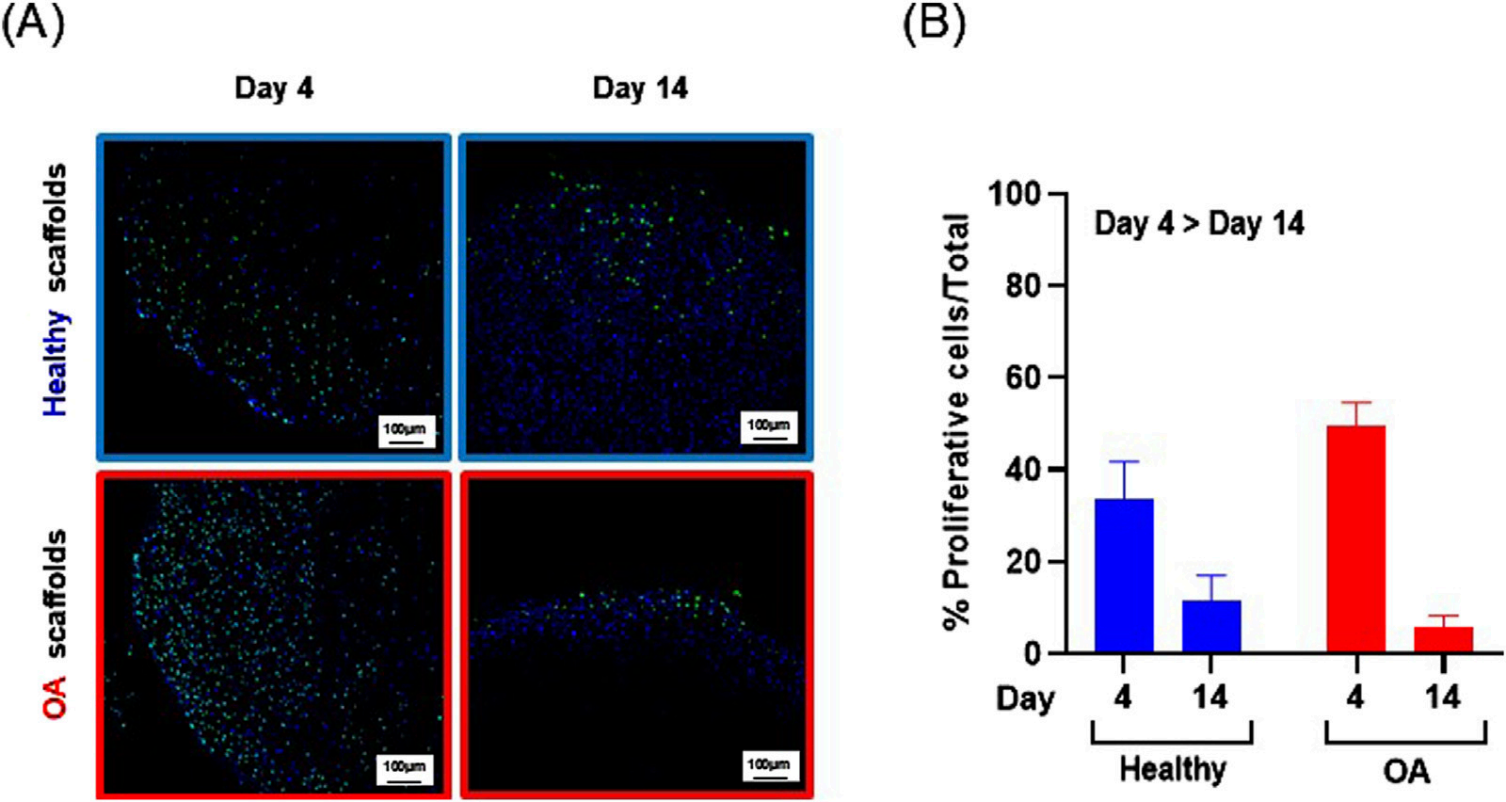
**(A)** Representative images of Edu+ (green) cells and total (blue) human meniscus cells seeded on healthy and OA scaffolds. **(B)** Percent proliferative cells per total cells on healthy and OA scaffolds. Data is expressed as the mean +SEM for all groups.

Actin immunostaining revealed the initial spherical shape of the human meniscus cells on both scaffold types at 1 and 3 h after seeding (Figure 5). For healthy scaffolds, the cells remained rounded at 6 h, while on the OA scaffolds the cells began to elongate. By 24 h, the cells appeared fibroblastic with cellular processes on both the healthy and OA scaffolds. Meniscus cells showed approximately 90% viability within both the healthy and OA scaffolds on both days 3 and 7 with no detectable differences between the scaffolds (Figures 6A,B). In both healthy and OA scaffolds, meniscus cells were more proliferative at day 4 than day 14 (Figures 7A,B, *p* < 0.05). There were no detectable differences in cellular proliferation between scaffold types.

### 3.4 Biochemical changes in meniscus cell seeded scaffolds

**FIGURE 8:**
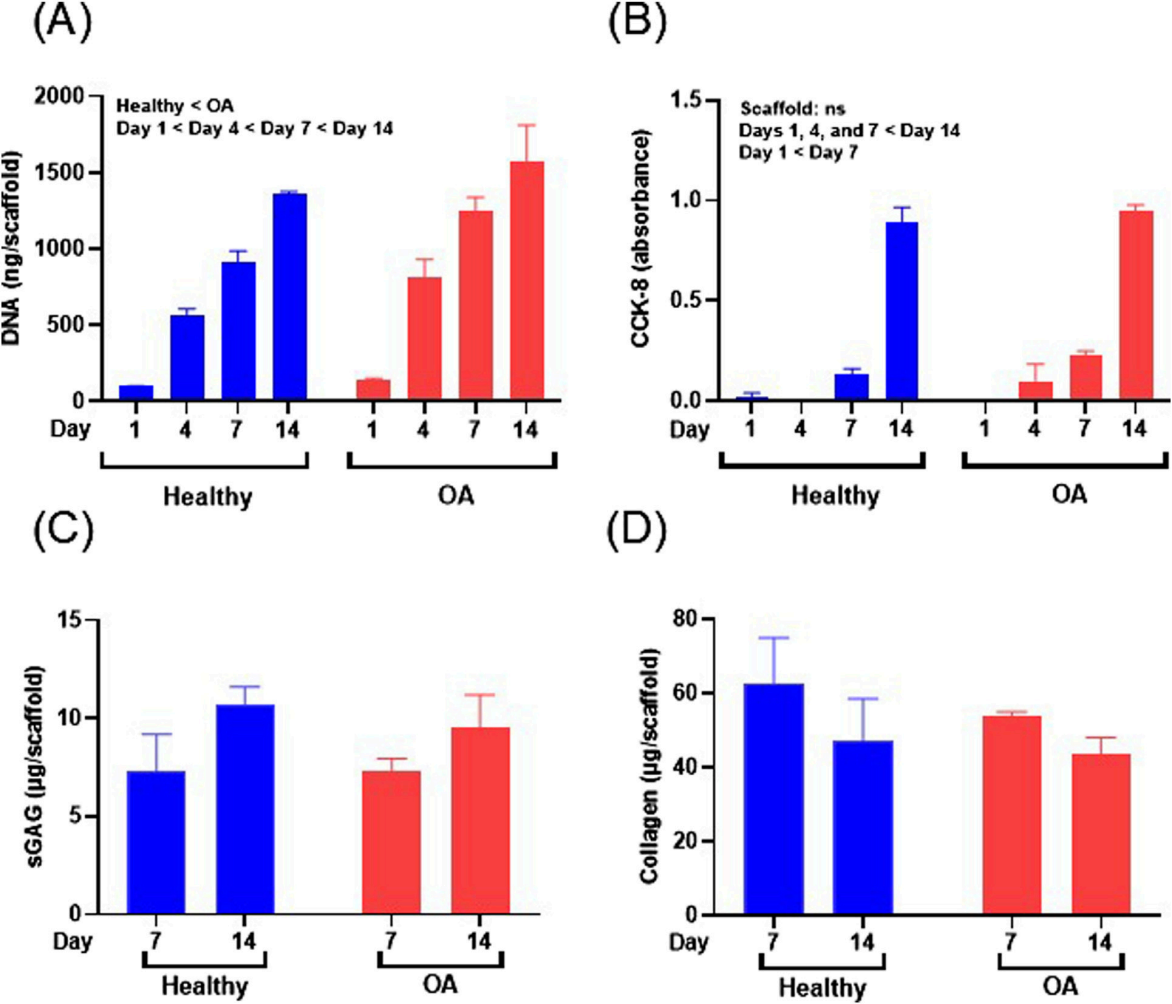
**(A)** DNA content, **(B)** CCK-8, **(C)** sGAG, and **(D)** collagen content of human meniscus cell-seeded healthy and OA scaffolds. Data are expressed as the mean +SEM for all groups.

The DNA content of meniscus cell-seeded healthy and OA scaffolds was significantly increased over 14 days (Figure 8A, *p* < 0.0001). Overall meniscus cell-seeded OA scaffolds contained higher DNA content than healthy scaffolds (*p* < 0.05). Generally, CCK-8 measurements, indicative of viable cell number, significantly increased over the 14 days of culture for both scaffolds (Figure 8B, *p* < 0.0001). However, there were no detectable differences in CCK-8 between OA and healthy scaffolds. sGAG content trended towards an increase from days 7–14 for both meniscus cell-seeded scaffold types (Figure 8C). There were no detectable differences in collagen content between scaffold types or over time in meniscus cell-seeded scaffolds (Figure 8D).

### 3.5 Meniscus cellular responses in the *ex vivo* defect model

**FIGURE 9:**
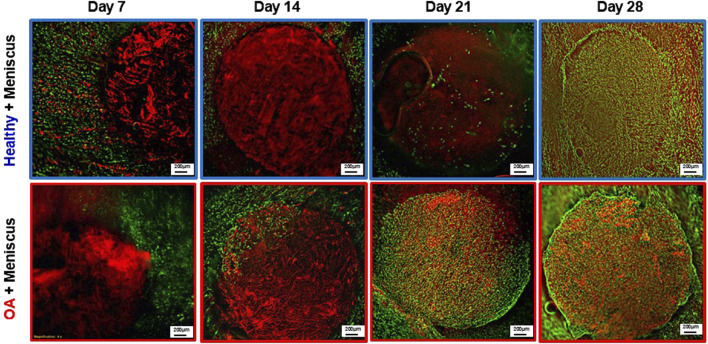
Representative fluorescent images of the Healthy scaffold + Meniscus and OA scaffold + Meniscus in the *ex vivo* defect model. Live cells (green) and matrix (red) are stained.

Fluorescent imaging on day 7 demonstrated that cells were located throughout the meniscus tissue outer ring but not in the healthy and OA scaffold inner cores (Figure 9). On day 14, while no cells were detectable in the healthy scaffolds, there were meniscus cells that had migrated from the outer ring into the edge of the implanted OA scaffold. On day 21 few cells had migrated into the healthy scaffolds but there were abundant meniscal cells distributed throughout the OA scaffolds. By day 28, both scaffold groups showed cellular infiltration throughout the core and at the interface between the outer ring and inner core. However, there was greater visible cellular bridging between the inner core scaffolds and the outer ring meniscus tissue in the Healthy + Meniscus samples than the OA + Meniscus samples.

### 3.6 Biochemical and shear strength evaluation in the *ex vivo* defect model

**FIGURE 10:**
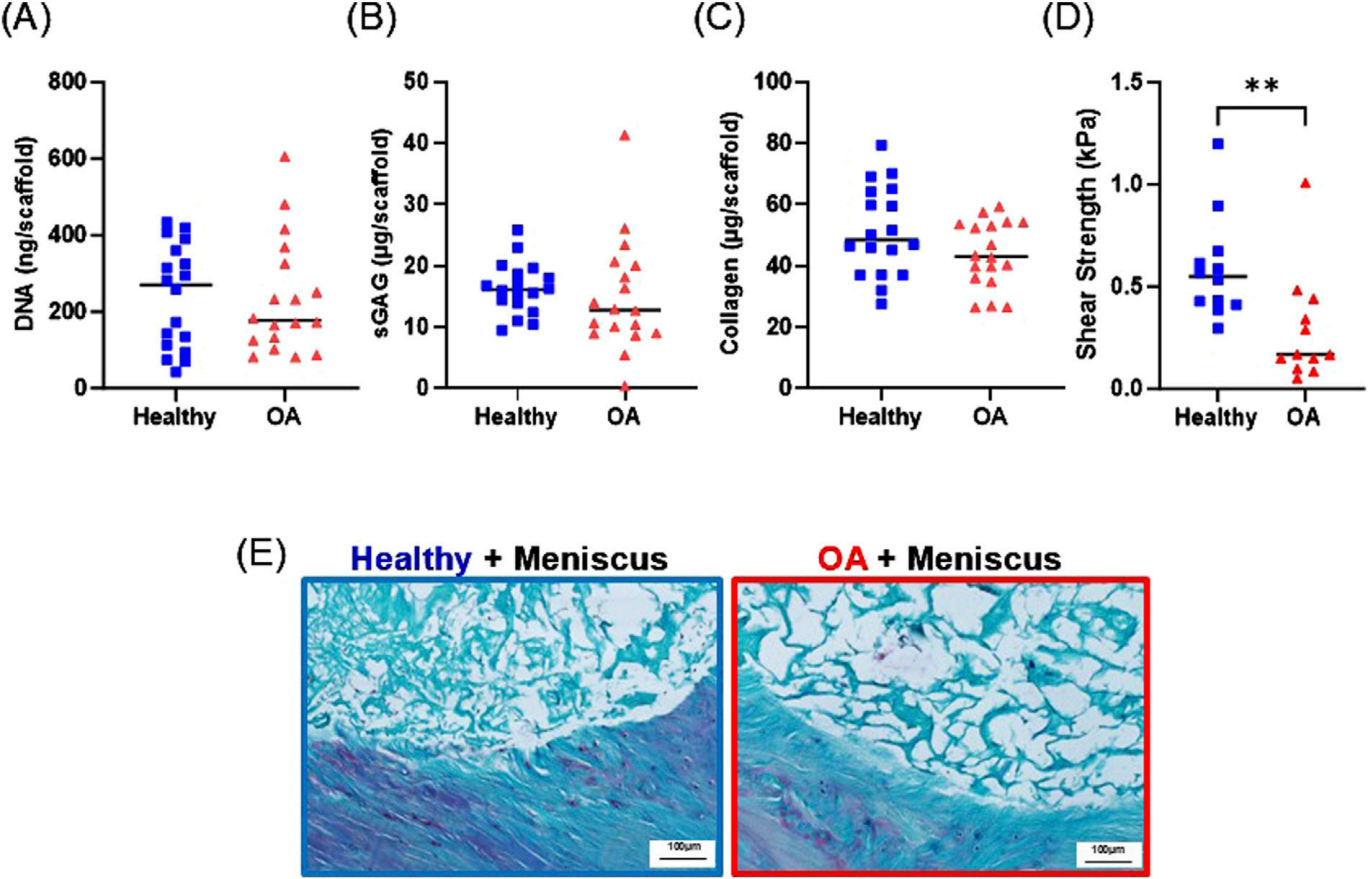
Day 28 **(A)** DNA, **(B)** sGAG, and **(C)** collagen content of healthy and OA scaffolds from the *ex vivo* meniscus defect model. **(D)** Shear strength of repair of the meniscus defect model. **p < 0.005. The mean is indicated by a line for each group. **(E)** Representative histological staining of the interface of the meniscus defect model explants. Sections were stained with Safranin-O (red: proteoglycans), fast green (blue: collagen), and hematoxylin (black: nuclei).

After 28 days of culture in the *ex vivo* repair model, there were no detectable differences between the DNA (Figure 10A), sGAG (Figure 10B), or collagen (Figure 10C) content between the healthy and OA scaffolds. However, the shear strength of repair was significantly lower in the defects filled with OA scaffolds compared to defects filled with healthy scaffolds (Figure 10D, *p* < 0.005).

### 3.7 Histological analysis of the *ex vivo* defect model

Histological analysis revealed that both the healthy and OA scaffolds integrated with the surrounding meniscus tissue (Figure 10E). While proteoglycan staining was detectable in the surrounding meniscus tissue, the healthy and OA scaffolds predominately stained for collagen. Both scaffold types were porous; however, there was more collagenous matrix present in the healthy scaffolds compared to the OA scaffolds.

## 4 Discussion

To our knowledge, this is the first study to compare the biocompatibility and functionality of healthy and OA MDM scaffolds that have potential for clinical practice as a cell-free tissue engineering strategy. Overall, we found that both healthy and OA scaffolds are biocompatible and promote cellular infiltration and growth. However, there was a significant difference in the functionality of the healthy and OA scaffolds based on the shear strength of repair, which quantifies the amount of healing between the meniscus tissue and the MDM scaffolds. Importantly, the shear strength of repair was reduced in meniscus defects containing OA scaffolds compared to healthy scaffolds, suggesting that the healthy scaffolds are promising for meniscus tissue engineering. In conclusion, we have shown that healthy human allograft tissue is a useful source for generating MDM scaffolds that can support cellular growth, ECM production, and *ex vivo* integrative repair of the meniscus, highlighting their suitability for future *in vivo* investigations.

In this study, we initially compared healthy and OA meniscus tissue. As expected (Pauli et al., 2011; Waldstein et al., 2016), OA tissue had higher gross grading scores than healthy tissue due to ECM disruption and meniscus tears. Furthermore, OA tissue contained regions of hypercellularity, which was consistent with the higher DNA content in the OA scaffolds prior to decellularization. Histologically, there were differences in Safranin-O staining in healthy *versus* OA tissue, but this difference was not statistically significant in the biochemical analysis of the MDM scaffolds. Much of the damage in the OA tissues was localized to the inner zone or resulted in the loss of the inner zone of the meniscus. Thus, due to the challenges of reproducibly identifying the inner zone and having enough remaining inner zone tissue for scaffold preparation, we used the whole meniscus to fabricate the scaffolds. It would be interesting to investigate the effects of zonal differences between healthy and OA scaffolds in the future.

The overall goal of this study was to compare healthy and OA scaffolds, but we were unable to remove age as a biological variable due to the patient populations. The OA tissues came from older donors than the healthy tissues. In this study, we cannot determine what differences in the scaffolds are specifically due to age-related changes *versus* osteoarthritis-related changes. Regardless, we were able to directly compare scaffolds generated from both healthy and OA meniscus tissues and show that there are functional differences between these scaffolds for meniscus tissue engineering applications.

To facilitate clinical translation of tissue engineering scaffolds, decellularization is necessary to reduce potential immunologic responses (Ahmed et al., 1983). The ideal decellularization is achieved when nucleic acid content is reduced, and the quantity and structure of ECM components are retained. In our prior work (Ruprecht et al., 2019; Lyons et al., 2019), we relied on loss of DNA content throughout cell culture; however, this prevents scaffolds from being implanted without prior culture. In this study, a new protocol was developed for the fabrication of decellularized MDM scaffolds from healthy and OA human menisci. Our decellularization method used a combined physical and enzymatic approach, which was effective at reducing the DNA content and retaining the majority of the major ECM components for both healthy and OA scaffolds. Following decellularization, on average each scaffold contained approximately 0.03 µg DNA/mg, which is a lower DNA concentration than several commercially available products used in patients, including MatriStem (1.56 μg/mg), which is a urinary bladder matrix, small intestinal submucosa (1.46 μg/mg), and XenMatrix (0.06 μg/mg), which is a dermis tissue (Huleihel et al., 2016). While the majority of sGAGs are preserved during our scaffold fabrication process, similar to prior studies (Rothrauff et al., 2017; Visser et al., 2015; Wu et al., 2015; Monibi and Cook, 2017), we experienced some loss of proteoglycans during our decellularization. Prior work has also revealed alterations in collagen content due to decellularization (Kasravi et al., 2023) but this was retained in both our healthy and OA MDM scaffolds.

Our *in vitro* results revealed that primary human meniscus cells attached, proliferated, were metabolically active, and retained ECM on both healthy and OA scaffolds for up to 14 days. Interestingly, the cellular responses were more promising on the OA scaffolds. Meniscus cells were able to spread into a fibroblastic morphology more quickly on the OA scaffolds compared to healthy scaffolds. This suggests that the OA scaffolds provide more adhesion sites and/or signaling cues for the cells, enhancing cell-biomaterial interactions (Goyal et al., 2017). Given that the cells were isolated from OA meniscus tissues, these cells may be primed for attachment to the adhesion sites in the OA scaffolds. Consistent with this difference in attachment, DNA content was higher in OA cell-seeded scaffolds throughout *in vitro* culture. Despite this observation, DNA content and CCK-8 measurements significantly increased over the 14 days in culture for both the healthy and OA scaffolds. In addition, there was high meniscus cell viability over time on both healthy and OA scaffolds. However, there was a decrease in the percent proliferative cells from day 4 to day 14 in both healthy and OA scaffolds. Notably, the proliferative cells were detected throughout the scaffolds on day 4; however, they were predominantly localized to the edges of the scaffolds on day 14. The change in location and number of proliferative cells over time is likely driven by cellular crowding within the interior of the scaffolds over time, resulting in reduced overall proliferation of the cells. Furthermore, ECM cues that drive proliferation (Sohrabi et al., 2023) may be less accessible at later times in culture. However, cellular growth and metabolic activity over time highlight the biocompatibility of the scaffolds. In future studies, it would be interesting to assess the gene expression profiles of meniscus cells on the scaffolds to understand transcriptomic changes in response to the healthy and OA scaffolds.

Both healthy and OA cell-seeded scaffolds produced similar amounts of sGAG throughout *in vitro* culture. However, there was an initial loss of GAG content that was likely due to leaching of the proteoglycans into the culture media from the scaffolds, which is similar to the phenomenon that is observed in meniscus explant culture (McNulty et al., 2010; Nishimuta and Levenston, 2012). Our prior work has shown that cross-linking of the MDM scaffolds can help to preserve the sGAG content (Lyons et al., 2019; Ruprecht et al., 2019) and may be useful to enhance retention of scaffold sGAGs in future studies. However, the sGAG content was trending towards an increase from days 7–14, indicating that the meniscus cells were able to synthesize new sGAGs that were incorporated into the scaffolds. These findings are similar to other work using PCL-meniscus ECM based hydrogel hybrid scaffolds or PCL nanofibrous scaffolds seeded with bovine meniscus cells, which showed a gradual increase in sGAG content over 14 days (Chen et al., 2019; Xia et al., 2021). Most promising is that in both healthy and OA scaffolds, meniscus cells secreted sGAGs that were retained during culture, which is an important feature for meniscus repair and regeneration.

The collagen content of both the healthy and OA cell-seeded scaffolds remained stable over the 14 days in culture. This data is consistent with another study that evaluated collagen content in meniscus cell-seeded ECM-PCL nanofibrous scaffolds after 2 weeks (Xia et al., 2021). Overall, both heathy and OA scaffolds can support collagen maintenance and/or production during culture.

Healthy and OA scaffolds showed nearly equivalent biochemical content but vastly different integrative shear strength of repair between the scaffold and native meniscus tissue in the *ex vivo* meniscus defect model. In native meniscus tissue, the dense collagen network is a barrier for cell migration to sites of meniscus injury (Qu et al., 2019; Qu et al., 2018). In our previous work, we showed that fabrication of the scaffolds with MDM powder leads to a porous structure that facilitates meniscus cell migration into the MDM scaffolds from the porcine meniscus tissue (Ruprecht et al., 2019; Lyons et al., 2019). We took advantage of this successful approach to compare human meniscus cell responses to healthy and OA scaffolds in a human *ex vivo* meniscus defect model. Cells from the meniscus tissue were initially able to migrate into the OA scaffolds more rapidly than the healthy scaffolds, suggesting that the OA scaffolds provide more chemotactic factors and/or adhesion sites (Goyal et al., 2017) to attract and adhere native meniscus cells. However, in both scaffold types, meniscal cells were viable over the 4 weeks in culture and native cells migrating from the surrounding meniscus tissue filled the interface and scaffolds. In our previous work, the addition of exogenous cells and scaffold cross-linking were not necessary to promote meniscus tissue repair (Ruprecht et al., 2019); therefore, we employed a cell-free approach and used non cross-linked scaffolds in this study. In this human *ex vivo* model system, without exogenous growth factors, both healthy and OA scaffolds promoted cellular migration into the scaffolds. However, the shear strength of repair was significantly lower in defects containing the OA scaffolds compared to the healthy scaffolds. This reduced shear strength in the OA scaffolds may be due to tissue health and/or age-related changes in the matrix integrity and composition of these scaffolds. While biochemically the healthy and OA scaffolds appeared similar, grossly and histologically it is clear that the OA tissue was more degraded and contained less proteoglycans. Furthermore, there are likely alterations in many other biochemical components in the OA tissue and scaffolds that were not quantified in this study. Additionally, the healthy scaffolds in the meniscus defect model showed more collagenous matrix histologically, which likely contributed to improved repair strength. In the future, proteomic analyses will be necessary to assess the compositional differences between the healthy and OA MDM scaffolds to determine the biochemical changes that may affect the functionality of the scaffolds.

A higher shear strength of repair is desired to improve clinical meniscus repair, as this indicates that the repair tissue can withstand greater mechanical forces. The repair strength of the healthy human MDM scaffolds in the *ex vivo* meniscus defect model was lower than previously observed for porcine MDM scaffolds in an *ex vivo* porcine meniscus defect model after 28 days (Ruprecht et al., 2019). This finding may be due to the use of human meniscus tissue from older patients with end stage osteoarthritis in this study. This older, OA tissue is likely not very metabolically active or amenable to repair. On the other hand, in our porcine work the tissue was from 2 to 3 year old pigs without remarkable OA changes (Ruprecht et al., 2019). Therefore, it would be interesting to see if the repair responses were improved in non-OA, acutely injured human meniscus tissue, which would likely be more amenable to meniscus repair. Additionally, a longer time in culture may increase cellular numbers in the scaffolds and improve ECM synthesis by these migrated cells (Hennerbichler et al., 2007b; Ionescu and Mauck, 2013). Furthermore, infiltration of the scaffolds by exogenous stem cells (Mandal et al., 2011b), structural modification of the scaffolds using cell adhesion motifs (Xu et al., 2020), cross-linking (Lyons et al., 2019), or coating with polymers or nanoparticle solutions (Li et al., 2021b) may further enhance cell migration and ECM synthesis but may impede translatability for future clinical applications. Additional studies will be necessary to determine if any of these modifications can further improve tissue repair.

In this study, we compared healthy and OA human MDM scaffolds and revealed that while OA scaffolds showed slightly more favorable cellular growth *in vitro*, both scaffold types were biocompatible and can provide a supportive environment for meniscal cells to attach, proliferate, and migrate but only the healthy MDM scaffolds could facilitate repair with native meniscus tissue. Therefore, healthy human allograft tissue is a useful source for generating MDM scaffolds that can support cellular growth, ECM production, and *ex vivo* integrative repair of the meniscus. Our findings highlight the suitability of the healthy MDM scaffolds for future testing in *in vivo* preclinical models for meniscus repair.

## Data Availability

The original contributions presented in the study are included in the article/supplementary material, further inquiries can be directed to the corresponding author.
